# A Framework and Methodology for Navigating Disaster and Global Health in Crisis Literature

**DOI:** 10.1371/currents.dis.9af6948e381dafdd3e877c441527cba0

**Published:** 2013-04-04

**Authors:** Jennifer L. Chan, Frederick M. Burkle

**Affiliations:** Emergency Medicine, Northwestern University/Harvard Humanitarian Initiative, Chicago, Illinois, USA; Harvard Humanitarian Initiative, Harvard School of Public Health, Harvard University, Cambridge, Massachusetts, USA

## Abstract

Both ‘disasters’ and ‘global health in crisis’ research has dramatically grown due to the ever-increasing frequency and magnitude of crises around the world. Large volumes of peer-reviewed literature are not only a testament to the field’s value and evolution, but also present an unprecedented outpouring of seemingly unmanageable information across a wide array of crises and disciplines. Disaster medicine, health and humanitarian assistance, global health and public health disaster literature all lie within the disaster and global health in crisis literature spectrum and are increasingly accepted as multidisciplinary and transdisciplinary disciplines. Researchers, policy makers, and practitioners now face a new challenge; that of accessing this expansive literature for decision-making and exploring new areas of research. Individuals are also reaching beyond the peer-reviewed environment to grey literature using search engines like Google Scholar to access policy documents, consensus reports and conference proceedings. What is needed is a method and mechanism with which to search and retrieve relevant articles from this expansive body of literature. This manuscript presents both a framework and workable process for a diverse group of users to navigate the growing peer-reviewed and grey disaster and global health in crises literature. Methods: Disaster terms from textbooks, peer-reviewed and grey literature were used to design a framework of thematic clusters and subject matter ‘nodes’. A set of 84 terms, selected from 143 curated terms was organized within each node reflecting topics within the disaster and global health in crisis literature. Terms were crossed with one another and the term ‘disaster’. The results were formatted into tables and matrices. This process created a roadmap of search terms that could be applied to the PubMed database. Each search in the matrix or table results in a listed number of articles. This process was applied to literature from PubMed from 2005-2011. A complementary process was also applied to Google Scholar using the same framework of clusters, nodes, and terms expanding the search process to include the broader grey literature assets. Results: A framework of four thematic clusters and twelve subject matter nodes were designed to capture diverse disaster and global health in crisis-related content. From 2005-2011 there were 18,660 articles referring to the term [disaster]. Restricting the search to human research, MeSH, and English language there remained 7,736 identified articles representing an unmanageable number to adequately process for research, policy or best practices. However, using the crossed search and matrix process revealed further examples of robust realms of research in disasters, emergency medicine, EMS, public health and global health. Examples of potential gaps in current peer-reviewed disaster and global health in crisis literature were identified as mental health, elderly care, and alternate sites of care. The same framework and process was then applied to Google Scholar, specifically for topics that resulted in few PubMed search returns. When applying the same framework and process to the Google Scholar example searches retrieved unique peer-reviewed articles not identified in PubMed and documents including books, governmental documents and consensus papers. Conclusions: The proposed framework, methodology and process using four clusters, twelve nodes and a matrix and table process applied to PubMed and Google Scholar unlocks otherwise inaccessible opportunities to better navigate the massively growing body of peer-reviewed disaster and global health in crises literature. This approach will assist researchers, policy makers, and practitioners to generate future research questions, report on the overall evolution of the disaster and global health in crisis field and further guide disaster planning, prevention, preparedness, mitigation response and recovery.

## INTRODUCTION

Over the past four decades disaster and global health in crisis research has grown tremendously and this growth is reflected in the rapid increase in peer-reviewed literature. This is partly due to the increasing number and size of disasters. In 2011, there were 332 natural disasters with over 244.7 million victims.^1^ According to UNHCR, over 4.3 million people were newly displaced in 2011, mostly due to conflict.^2^ The multidisciplinary and transdisciplinary field of practitioners, policy-makers, and researchers, many who are experts in sociology, information sciences, law, economics and ethics, just to name a few, continue to push boundaries and challenge this quickly changing complex space. What results is an ecosystem of health professionals and a growing number of cross-breeding experts that are defining the multidisciplinary and transdisciplinary nature of disaster and global health in crisis fields with their research and writings.^3^ The peer-reviewed literature has reflected this change with a massive explosion of information across many journals. The demand to access and use the literature for policymaking, program decisions, and research continues to grow. Whereas the sheer volume and growth of peer-reviewed literature in disasters and global health in crisis can be viewed as a testament to the value and relevance of the field, what results is a deluge of knowledge which for practitioners, policy makers and researchers alike may prove unmanageable to access.

Many disaster and global health in crisis research studies describe searching databases by event types or a specific disaster or crisis. Studies by Kelen and Smith both search the literature to determine which journals frequently publish disaster health and event-specific literature respectively.^4^^,^ 5 A recent study showed that practitioners seek evidence-based information, but view the evidence within the context of “usefulness”.^20^ This was cited as relevant to specific disaster contexts and health systems research in addition to clinical trials. The United States Department of Health and Human Services Department of Information Management Research Center has made efforts to improve the access to health information during all phases of the disaster management cycle including an online glossary of disaster terms.^6^ The Evidence Aid Project, an initiative of the Cochrane Collaboration, aims to collect and freely share “reliable, up-to-date evidence on interventions that might be considered in the context of natural disasters and other major healthcare emergencies.”^21^

PubMed, a free online database from the National Library of Medicine is recognized as one of the major English language health and medicine databases. With over 22 million citations PubMed is a hierarchical search engine that uses keywords and medical search terms (MeSH). To date, PubMed still serves as one of the primary databases for which to search the peer-reviewed literature for health and medicine. It is also the database used for published literature reviews such as the annual Global Emergency Medicine Literature Review and many published papers on disaster medicine, public health crises and global health. 4 ^,^ 7 ^,^ 8

In 2004 Google launched Google Scholar, a web-crawling free and online search engine. Strong in the health, science and engineering disciplines, Google Scholar searches have the potential to expand the search environment for users interested in global health and crisis literature.^8^ Google Scholar provides added value to users interested in searching multidisciplinary and transdisciplinary topics and accessing the grey literature (e.g., governmental documents, United Nations agency reports, conference proceedings, consensus reports and books.)^10^ It has also been described as a search engine that serves as a quick and easy starting point for literature exploration and provides search returns of core documents in that topic domain.^9^

These powerful online search engines and databases open doors to accessing thousands of articles. However, there has yet to be an article that creates a ‘roadmap’ or process for users to approach the peer-reviewed literature from different angles or a definition of disaster and global health in crisis that is equally inclusive. While a researcher may seek to perform a systematic review of national preparedness plans for emergency vaccination programs, a health practitioner from a non-governmental organization may search the literature for the impact of community-based vaccination programs to help guide their programs. It is known that clinicians often face obstacles when searching the literature, including challenges in determining search order, and filtering and determining the adequacy of a search.^11^ It is likely that these diverse stakeholders in the global health and disaster community face similar, if not greater, challenges.

A flexible framework and practical process for navigating disaster and global health in crisis literature is needed. It should be approachable by different stakeholders for different purposes. While academics may search the literature to investigate emerging areas of research, policy makers may seek literature that spans two disciplines to inform legislation. Practitioners may look for evidence-based practices to guide programming or clinical practice. The framework and process should aim to prevent the ‘silo-ing of information’ and push forward or reconfigure knowledge sharing. This framework falls in line with published literature that promotes interdisciplinary engagement and an “integrative expertise.”^3^

This manuscript presents a framework, process, and methodology for searching, collating, and navigating the broad base of growing disaster and global health in crises literature with a primary focus on peer-reviewed literature. It presents the rationale for a framework that acts as a roadmap to search disaster and global health in crisis literature. A hierarchical classification scheme of structured clusters and nodes (italics), MeSH terms (brackets), and free text terms (single quotes) creates a taxonomy that is specific to PubMed. Google Scholar is also used to transition from a hierarchical search process to a network search structure that allows for a comparative and complementary approach to searching the literature. Examples of how to use this framework is presented in tabular and matrix format exemplifying the process with which researchers, policy makers and practitioners can navigate a database of thousands of disaster and global crisis articles. Lastly, future opportunities, current challenges, and next steps are discussed for the proposed framework and search process.

## METHODOLOGY

Frameworks can provide a plan and strategy for organizing large volumes of information. In this context we include disaster and global health in crisis literature. Within this organized search structure is a taxonomy of layered terms. For global health, disasters and crisis, the taxonomy should reflect health issues, health systems, disaster and crisis event types, and study disciplines that focus on these issues. The proposed goal of this framework is to assist researchers, practitioners, and policy makers to search and explore the literature. In this process one should be enabled to learn more about this interdisciplinary and transdisciplinary field, understand where knowledge gaps exist, and be informed of new frontiers for research, practice, and policymaking. A structured but inclusive and flexible framework is necessary to match the quickly evolving field.

Eight textbooks, two peer-reviewed articles, two disaster manuals, and unpublished documents were reviewed from sources considered reflective of expert knowledge. This method of reviewing and retrieving terms from relevant documents has been performed in other published studies.^12^ Terms from tables of contents and glossary of terms from published literature were collected and collated.^13^^,^ 14 ^,^ 15 (Table 1) When terms or phrases occurred at least 2-3 times through the curation process the terms were included in the creation of the final 84 search terms.

**Table 1: Document sources for search terms:** 

	Title	Authors/Editors	Publisher	Location
1	Basic Disaster Life Support Version 3.0 Course Manual	American Medical Association	AMA	USA
2	Core Disaster Life Support Manual, Version 3.0	American Medical Association	AMA	USA
3	Emergency Public Health: Preparedness and Response	Kapur GB, Smith JP	Jones & Bartlett Learning	USA, Canada, UK
4	Disaster Medicine	Hogan DE, Burstein JL	Lippincott Williams & Wilkins	USA
5	Disaster Medicine	Ciottone GR	Mosby	USA
6	Oxford American Handbook of Disaster Medicine	Partridge RA, Lawrence P, Marcozzi D	Oxford University Press	UK
7	Koenig and Schultz's Disaster Medicine: Comprehensive Principles and Practices	Koenig KL, Schultz CH	Cambridge University Press	UK
8	Public Health Management of Disaster Medicine	Landesman LY	American Public Health Association	USA
9	Humanitarian crises: the medical and public health response	Leaning J, Briggs S, Chen LC	Harvard University Press	USA
10	H.E.L.P Public health course in the management of humanitarian aid	Perrin P	ICRC	Switzerland
11	Trend analysis of disaster health articles in peer-reviewed publications pre- and post-9/11. *Am J Disaster Med.* 3(6):369-76	Kelen G, Sauer LM	Weston Medical Publishing	USA
12	Three decades of Disasters: A Review of Disaster-Specific Literature from 1977 -2009. *Prehosp Disaster Med. *2009;24(4)	Smith E, Wasiak J, Sen A, Archer F, Burkle FM	Cambridge University Press	USA/UK

A total of 143 terms or phrases were curated from documents. Eighty-four terms were selected that occurred in three or more documents and organized into four clusters (Table 2). When terms were synonymous with PubMed medical subject headings (MeSH), the MeSH term was chosen. The four clusters and nodes were created by the curation process of the authors who reviewed over 200 terms and phrases.

**Table 2: Cluster, Nodes and Terms (*free text terms are italicized for clarity):** 

	Nodes	Example MeSH and Free Text Terms
Cluster 1 Meta-topics	General Terms	crisis, emergency, medicine, EMS, relief work, *humanitarian*
	Disciplines	epidemiology, health system, population, public health, mental health
	Adjunct Discipline	ethics, policy
Cluster 2 Operations	Communication & Management	communication, management, information system, disaster planning, health planning
	Training & Education	training support, education, training program
	Learning & Evaluation	evaluation studies as a topic, learning, health impact assessment, needs assessment, outcome assessment, standards of care, *surveillance, monitoring,* *evaluation, impact*
Cluster 3 Administrative Activities & Systems	Administrative Levels	federal government, state government, state health planning agencies, local government, community health centers, community health planning, community health services, national health program regional health planning, regional medical programs.
	Special Groups	elderly/aged, vulnerable populations, refugees, children, *special populations*
	Sites of Care	hospital, health facility, EMS, *alternate care*
Cluster 4 Events & Phases	Events	epidemic, pandemic, terrorism, war, mass casualty incidents, earthquakes, floods, hurricanes, tornadoes, tsunamis,*chemical disasters, natural disaster, extreme temperature, industrial/technical disaster, nuclear disaster, complex emergency, conflict, biological disaster, wildfires*
	Activities	triage, surge capacity, decontamination, emergency shelter,*evacuation, shelter*
	Phases	emergency preparedness, rehabilitation,prevention, *preparedness, mitigation, disaster risk reduction, response, recovery*

**Diagram of four clusters and twelve nodes, and search terms:**
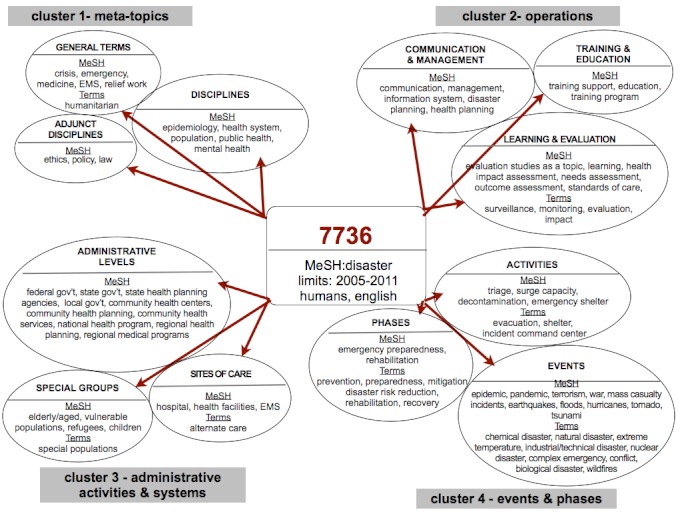

Cluster 1, titled *meta-topics*, includes the most general disaster and crisis terms, academic disciplines most tightly related to the field, and adjunct disciplines. Cluster 2, *operations*, includes terms related to disaster and crisis operations including communications, management, education, and assessments and evaluation. Cluster 3 reflects *administrative activities and systems*. Lastly, Cluster 4 includes *events/phases*. The terms that lie within each cluster are called nodes as they are inter-related to one another within that specific cluster. (Figure 1) For example, Cluster 4 has three nodes: *events*, *phases*, and *activities*. Events such as hurricanes and bioterrorist events are all disaster types and each event type can span parts of the disaster cycle including mitigation, preparedness, response and recovery.

The terms organized within each *node* and *cluster* are derived from two sources: 1) medical subject headings [MeSH] from the National Library of Medicine’s PubMed database and 2) ‘free text terms’ (Figure 1). There are 26,853 MeSH terms in the PubMed database and every article is indexed using these terms, which allows articles to be searched by database users. Using MeSH terms to query the PubMed database has been shown to have higher precision.^12^ The use of ‘free text terms’ has been shown to improve article retrievals.^12^ ^,16 Within the proposed framework using free text and MeSH terms as well as applying the matrix aim to enable the user to determine what level of sensitivity or specificity they would like to pursue for their search based upon their needs. ‘Free text terms’ were added to this taxonomy to capture new terms, not currently in the MeSH thesaurus, that reflect the quickly evolving field of global health, disasters, and crisis.16^

Crossing search terms MeSH and ‘free text terms’ using Boolean operators were used to filter thousands of articles into a manageable set of return articles. This approach has been shown in a previous study to increase the sensitivity and specificity of search returns.^17^ Representing search results in a tabular or matrix format allowed PubMed database users a visually practical format for which they may 1) extract relevant article sets for specific research or educational purposes 2) understand the current state of published research within this specific node and 3) identify gaps in research that could be further explored. In a matrix depicting the number of article returns in a row and column format can visually represent an entire cluster.

## RESULTS

The proposed disaster and crises framework for searching the PubMed literature database was used to search for articles from January 1^st^, 2005 to December 31^st^, 2011. Before using the framework a basic search for articles with the free text term ‘disaster’ was searched in PubMed resulting in 18,604 article returns. Restricting articles to human subjects, English language, and the [disaster] MeSH term resulted in 7,736 articles. A standard search of this kind would result in an otherwise restrictive and unmanageable subset of literature.

When applying the proposed framework to the 7,736 articles using the cluster, node, and terms framework articles can be reorganized and further filtered. Searching within a cluster can be achieved using a matrix that crosses terms between nodes. Figure 2 depicts an 11 by 11 matrix of two nodes, *general terms* and *disciplines* in Cluster 1 *meta-topics*. If the major term [disaster] is crossed with the MeSH term [relief work], 900 articles are returned. Although this restricts the types of articles by approximately 9-fold it remains unmanageable. By further cross searching the literature with terms within each node and across nodes the search returns range from 1 to 3865 articles. The majority of articles exist within [demography], [public health] and [health systems]. Fewer articles are returned in the field of [mental health] but predominate with the terms [demography], [health systems] and [public health]. Crossing the search terms [disaster], and free text term ‘humanitarian’ and MesH keywords [health systems] there are 66 returned articles which can be reasonably reviewed by a user.

**11 by 11 Matrix of meta-topic cluster 1, including two nodes:**
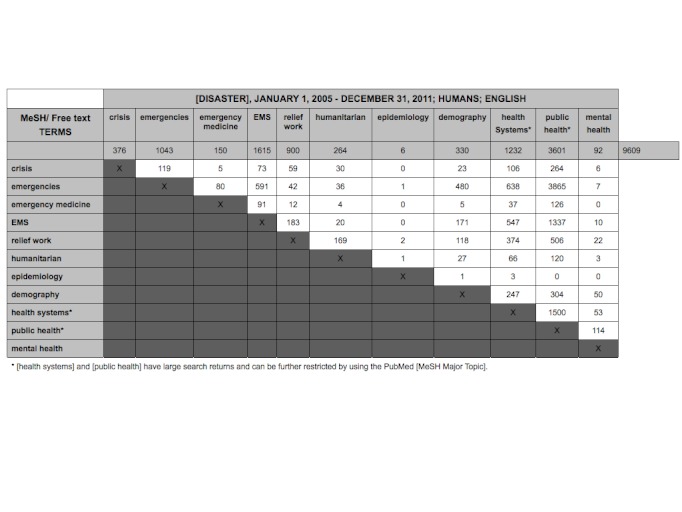

For users with a more specific area of interest, the tabular format can also show the distribution of terms within each node. When sorted by ascending or descending values it can elucidate which terms or related topics have large or small numbers of published literature. For* administrative activities and systems* cluster 3, terms within each node can be crossed with [disaster], producing three tables; one for each node in the cluster. The resulting tables are depicted in Figure 3.

**Example Tables from Cluster 3:**
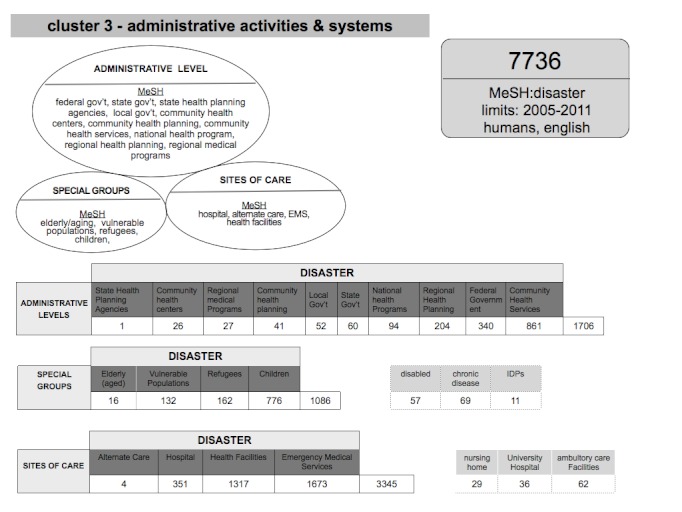

The *administrative activities and systems* node MeSH search terms were crossed with the term [disaster] with a total of 1706 article returns. The top two search returns came from crossing [disaster] with [community health services] (n=861) and [Federal Government] (n=340). There was only one return for [state health planning agency], but also 60 articles for [state government]. Results from the *special groups* node show that the largest body of literature within special groups is with the search terms [children] (n=776) and refugees (162). The smallest returns from all 1086 returns for this node were related to [elderly] (n=16). The terms within the *site of care* node resulted in 3345 articles. The majority of articles lie within the [EMS] and [health facilities] terms, and the term [alternate care] only returned four articles.

For searches that result in few PubMed returns searching Google Scholar can retrieve peer-reviewed literature in a broader array of journals while also retrieving related grey literature. The framework and above-described search terms can be applied to Google Scholar. According to Figure 3 that depicts a matrix of returns for two nodes within the first cluster, crossing the free text term ‘humanitarian’, and MeSH terms [disaster] and [mental health] in PubMed resulted in only three articles. Adding the term [relief] resulted in 25 articles. The same terms used in Google Scholar resulted in 65 total documents including books, peer-reviewed articles in non-health journals and United Nations reports. Nine of the returned articles were unique to the Google Scholar search. (Table 3) The remaining 56 articles were duplicative of the PubMed search process.

**Table 3: Unique Google Scholar Articles Compared to PubMed Search:** Search: mental health, disaster, crisis, global health international health, humanitarian relief.

Document Title	Type	Exists in PubMed?	Found in PubMed Search?	Open Access
Betancourt TS, et al. Building an evidence base on mental health interventions for children affected by armed conflict. *Intervention.* 2008;(6):39	Peer Reviewed Journal	yes	no	yes
Diaz JO, et al. *Advances in disaster mental health and psychological support.* New Delhi, India: Voluntary Health Association of India Press, 2006.	Book	no	no	yes
Budosan B, et al. "Evaluation of effectiveness of mental health training program for primary health care staff in Hambantota District, Sri Lanka post-tsunami." *The Journal of Humanitarian Assistance.* 2009	Peer Reviewed Journal	no	no	yes
Eriksson CB, et al. "Predeployment mental health and trauma exposure of expatriate humanitarian aid workers: Risk and resilience factors." *Traumatology *(2012).	Journal	no	no	no
Budosan B, et al. "After the wave: a pilot project to develop mental health services in Ampara district, Sri Lanka post-tsunami." *J Human Assist. *2007	Peer Reviewed Journal	no	no	yes
Ventevogel P, et al. "An intervention Special Issue Integrating mental health care into existing systems of health care: during and after complex humanitarian emergencies." *Intervention. 2011;*(9.30): 195-210.	Peer Reviewed Journal	no	no	yes
Saxena S et al. "Assessing mental health and psychosocial needs and resources: Toolkit for humanitarian settings." UNHCR	UN Agency Document	no	no	yes
Jenkins, R. "Supporting governments to adopt mental health policies." *World Psychiatry. *2003; (2.1):14.	Policy Report	yes	no	yes
Shah SA. "Ethical standards for transnational mental health and psychosocial support (MHPSS): Do no harm, preventing cross-cultural errors and inviting pushback." *Clinical Social Work Journal. *2011: 1-12.	Peer Reviewed Journal	no	no	no

When using PubMed to search for articles related to [alternate care] sites (or alternate sites of care) and [disasters] only four articles were returned. When using Google Scholar to search a similar set of terms it also returned articles with the following phrases ‘alternate care systems’ and the acronym ‘ASC’ expanding the search return. Applying the same framework to Google Scholar resulted in nine documents, the majority of which were neither archived in PubMed nor identified with PubMed search functions. (Table 4) There were two articles that existed in PubMed that were not retrieved by the PubMed search string but retrieved by Google Scholar. Boolean searches through Google Scholar may be better fit for variations in terminology.

**Table 4: PubMed vs. Google Scholar search return comparison for ‘disasters’ and ‘alternate (sites of) care’:** * Article found in GoogleScholar but not returned on the PubMed search, although they exist in PubMed

PubMed Search Results	Google Scholar Search Results *
search string- [disaster] and ‘alternate sites of care’	search string- intitle: "alternate care" AND disaster
Roszak AR, et al. Implications of the Emergency Medical Treatment and Labor Act (EMTALA) During Public Health Emergencies and on Alternate Sites of Care. Disaster Med Public Health Prep. 2009 Dec;3 Suppl 2:S172-5.	
Kost GJ, et al. Enhancing crisis standards of care using innovative point-of-care testing. Am J Disaster Med. 2011 Nov-Dec; 6(6):351-68.	* Chung S, et al. Pediatric Alternate Site of Care During the 2009 H1N1 Pandemic. Pediatric emergency care 27.6 (2011): 519-526.
Reilly MJ, et al. Utilizing paramedics for in-patient critical care surge capacity. Am J Disaster Med. 2010 May-Jun;5(3):163-8.	Cantrill SV, et al. Disaster alternate care facilities: Selection and operation. Rockville, MD: Agency for Healthcare Research and Quality. 2009: 152.
Hrdina CM, et al. The" RTR" medical response system for nuclear and radiological mass-casualty incidents: a functional TRiage-TReatment-TRansport medical response model. Prehosp Disaster Med. 2009 May-Jun;24(3):167-78.	Cantrill SV, et al. Disaster Alternate Care Facilities: Report and Interactive Tools. Rockville, MD: Agency for Healthcare Research and Quality. 2011 Publication No. 09-0062.
	* Kim CS, et al. Role of hospitalists in an offsite alternate care center (ACC) for pandemic flu. J Hosp Med. 2009 Nov;4(9):546-9.
	Hanfling D. "Alternate Care Systems: Stratification of Care." Institute of Medicine. 2009
	Reilly M. Creating Alternate Care Sites and Community-Based Care Centers for the Delivery of Medical Care During Public Health Emergencies. Prehosp Disaster Med 26.S1 (2011): s161-s161. (Abstract)
	Qureshi K. (A139) Profile of Likely Needs in a Disaster Alternate Site Care in Honolulu, Hawaii. Prehosp Disaster Med 26.S1 (2011): s39-s40. (Abstract)
	Fierro JM. Alternate care sites. California State University. 2010 (Dissertation).

Attempting to use Google Scholar for crossed search terms with larger PubMed returns results in an unmanageable number of returns. For example within Cluster 1, crossing the terms [disaster], [relief work] and [crisis] in PubMed, 59 articles are returned. Searching with the same terms in Google Scholar results in 2,210 returns. Searching a term within a node such as [federal government] and crossing it with [disaster] results in 340 articles in PubMed and 20,100 articles in Google Scholar.

## DISCUSSION

The multidisciplinary field of disaster and global health in crisis is a rapidly growing field of research and practice. And with this, there has been an explosion of literature creating an unmanageable body of information to search, retrieve and use. The proposed framework incorporates over 84 selected terms organizing them into clusters and nodes. The framework creates a structure with which to search the literature. The matrix and tabular formats enable a feasible process with which to retrieve a set of content-specific literature for review. When search returns yield a small number of articles, expanding the search to Google Scholar can provide added value by searching academic journals beyond PubMed database and also by including the grey literature.

**Limitations**

The proposed framework and process is not without limitations. The disaster and global health in crisis fields are rapidly changing and the cluster, nodes and terms may change over time. The current framework allows for interchangeable MeSH terms and the addition or replacement of free text terms. In addition, expert panels or additional research methods including the Delphi method or even natural language processing of documents could be employed to improve the selection of search terms and structuring of clusters and nodes. It is also possible for future iterations to include additional clusters and nodes when appropriate. For example, as seen in Figure 4, adding the MeSH search terms [disabled], [chronic disease] and free text term [Internally displaced population (IDPs)] result in a total of 137 articles. The search terms in the proposed framework preferentially use MeSH terms and assume that the MeSH terms are appropriately assigned and updated to best reflect terms in disaster and global health in crisis. While the majority of terms fit this assumption, examples such as the MeSH terms [demography] and [epidemiology] in cluster 1, Figure 2 appears to have a paucity of disaster and epidemiology literature and more demography literature. Two articles from humanitarian crisis and mortality literature were chosen and the indexing was checked revealing that there are variations in MeSH assignments.^18^^,^ 19One article had the MeSH term [epidemiology] assigned and neither had the free text terms ‘humanitarian’ or MeSH term [relief]. This classification variation was also found with natural disaster articles using epidemiological methods where the term [epidemiology] was not listed as one of the primary MeSH terms. Future research is necessary to better understand and validate the MeSH article indexing process which may potentially guide revisions to the National Library of Medicine PubMed indexing process and MeSH glossary of terms.

The framework of clusters, nodes and terms can be applied to other searchable databases with some revisions. The terms in this framework are based off of existing MeSH terms from the National Library of Medicine database. For other databases such as Psycinfo or EMBASE the terms would need to match those specific searchable keywords or have functions to search free text terms.

Google Scholar provides a complementary approach to searching the literature but can also pose significant limitations if the search methodology is not restricted.^9^ This is exemplified in the large search returns for general disaster and global health in crisis terms. While potentially helpful in tracking the total number of articles, it proves unmanageable with respect to accessing and reading the articles for decision-making purposes. The proposed framework and process provides a structured and restricted format for search returns. In addition exact comparisons between the search engine and PubMed database poses a challenge because the two systems are inherently different in structure and scope.^9^ Further investigation is needed to determine the best and most accurate methodology for comparing the two systems for rigorous comparison.

It is important to recognize that the ability to search a large body of literature using this framework and process does not always ensure that users can access the literature for knowledge, learning, and research. While some journals and grey literature are freely available for download, many peer-review articles are not open access and require institutional or personal subscriptions to access this knowledge-base. This is often a limiting factor for practitioners, some policy makers, and many researchers around the world. While outside the scope of this discussion, it is important to recognize this ongoing limitation and challenge.

## CONCLUSIONS

Despite the limitations discussed above, the framework and process to search the peer-reviewed and grey literature can assist a broad array of researchers, practitioners, and policy makers to advance the fields of disaster and global health in crisis. Specifically, researchers can explore the existing body of literature to identify gaps in scholarship and add to the body of literature on key issues within the field. Practitioners will learn from evidence-based research through reviewing content specific literature and improve decision-making and programming during future disasters. Policy makers can better use evidence-based literature to help inform legislation and future policy. And perhaps most importantly, the framework and process will help bring this multidisciplinary and transdisciplinary group closer together by engaging users in reaching toward content and literature among other disciplines, in health and beyond.

## COMPETING INTERESTS

The authors have declared that no competing interests exist.
